# Alterations in rice chloroplast integrity, photosynthesis and metabolome associated with pathogenesis of *Rhizoctonia solani*

**DOI:** 10.1038/srep41610

**Published:** 2017-02-06

**Authors:** Srayan Ghosh, Poonam Kanwar, Gopaljee Jha

**Affiliations:** 1 Plant microbe interactions laboratory, National Institute of Plant Genome Research, Aruna Asaf Ali Marg, New Delhi-110067, India

## Abstract

Sheath blight disease is caused by a necrotrophic fungal pathogen *Rhizoctonia solani* and it continues to be a challenge for sustainable rice cultivation. In this study, we adopted a multi-pronged approach to understand the intricacies of rice undergoing susceptible interactions with *R. solani*. Extensive anatomical alteration, chloroplast localized ROS, deformed chloroplast ultrastructure along with decreased photosynthetic efficiency were observed in infected tissue. GC-MS based metabolite profiling revealed accumulation of glycolysis and TCA cycle intermediates, suggesting enhanced respiration. Several aromatic and aliphatic amino acids along with phenylpropanoid intermediates were also accumulated, suggesting induction of secondary metabolism during pathogenesis. Furthermore, alterations in carbon metabolism along with perturbation of hormonal signalling were highlighted in this study. The gene expression analysis including RNAseq profiling reinforced observed metabolic alterations in the infected tissues. In conclusion, the present study unravels key events associated during susceptible rice-*R. solani* interactions and identifies metabolites and transcripts that are accumulated in infected tissues.

The sheath blight disease is one of the major diseases of rice and is reported from almost all rice cultivating countries in the world. The estimated yield loss due to this disease reaches up to 50% annually under favourable conditions^1^. Till date, no artificial or natural genetic variation is known for its complete resistance, making this disease more challenging to control. The disease resistance is thought to be a polygenic trait and some QTLs (quantitative trait loci) have been identified^2^,^3^,^4^. However usages of QTLs are limited in conventional disease resistance breeding programmes. Various control measure adopted for combating the disease includes agronomic practices such as crop rotation and usages of biological and chemical (fungicides) agents^5^,^6^,^7^,^8^,^9^. But none of them have proved adequate to control this disease.

The sheath blight disease is caused by a basidiomycete necrotrophic fungus, *Rhizoctonia solani* Kuhn (telemorph; *Thanetophorus cucumeris*) anastomosis group AG1-IA. In general *R. solani* strains demonstrate broad host range and cause disease on diverse plants including cereals, potato, bean, cotton, sugar beet, lettuce, melon, forest trees, and ornamental plants^10^,^11^. Also different strains demonstrate hyper variability in terms of its morphological and pathological attributes. The *R. solani* has been classified into 14 different anastomosis groups, i.e. AG-1 to AG-13 and AG-BI^12^,^13^,^14^,^15^. Recently draft genome sequences of AG1-IA (36.94 Mb encoding 10489 ORFs), AG1-IB (47.65 Mb encoding 12268 proteins), AG3 (51.7 Mb encoding 12726 proteins) and AG8 (39.8 Mb encoding 13964 proteins) have become publicly available^1^,^16^,^17^,^18^. Efforts through transcriptomics and comparative genomics approaches, are being made to understand the pathogenicity determinants of *R. solani*^1^,^16^,^18^,^19^.

Knowledge about the enigmatic necrotrophic interaction between *R. solani* and rice during establishment of sheath blight disease is very limited. Therefore, this study was conducted to understand the pathological, biochemical, and physiological alteration during pathogenesis of *R. solani* AG1-IA in susceptible rice cultivar PB1 (Pusa Basmati-1). The analysis reflected extensive anatomical and physiological alteration in host. GC-MS and RNA seq analysis delineated cascade of events associated with pathogenesis of *R. solani* in rice and identified molecular players involved in this process. Our analysis suggests modulation of host photosynthesis, respiration, phytohormonal signalling and secondary metabolism to be crucial for establishment of sheath blight disease.

## Results

### Dynamics of *R. solani* colonization during susceptible interaction in rice

**Figure 1:**
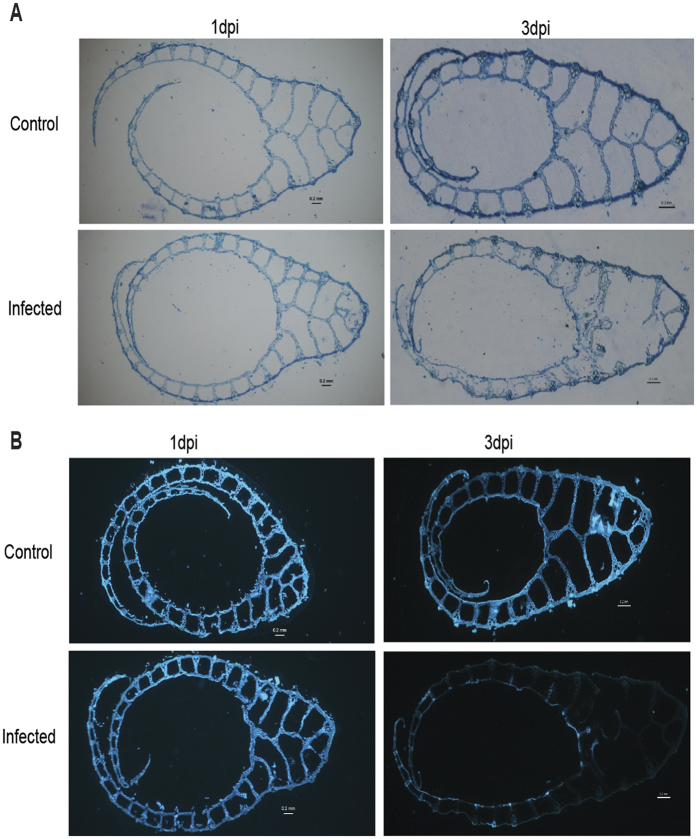
Distorted cellular anatomy of *R. solani* infected PB1 rice sheath. (**A**) T.S of toluidine blue stained rice sheaths. At 3dpi the infected sheaths demonstrated distorted cellular anatomy while no alteration was detected at 1dpi. (**B**) T.S of calcofluor white stained PB1 rice sheaths showing faint staining pattern at 3dpi. The data presented is representative of at least three independent biological replicates with at least 3 sheaths from different tillers being analyzed at each time points. Scale bar = 0.2 mm.

We had previously reported that a *R. solani* strain BRS1 belonging to AG1-IA anastomosis group causes characteristic sheath blight disease symptoms (i.e elliptical brown necrotic lesion) on different rice cultivars i.e *Oryza sativa* ssp. *indica* (PB1 and TETEP) and *O. sativa* ssp. *japonica* (TP309)^19^. Based upon disease severity index, PB1 and TP309 were susceptible while TETEP was partially resistant. In this study, we endeavoured to understand the infection processes of BRS1 on rice. Confocal microscopy of calcofluor white stained infected PB1 rice leaves revealed fungal mycelia growing parallel to rice veins (vasculature) at 1dpi. By 2dpi, extensive hyphal branching was observed while several infection cushions like structures overlapping with disease lesions were prominent during 3dpi (Fig. S1). To understand anatomical changes imparted by *R. solani*, the transverse section (T.S) of rice sheaths were studied through histological staining. Toluidine blue and calcofluor white staining revealed cellular disintegration of infected PB1 sheaths at 3dpi (Fig. 1A,B). Notably, neither toluidine blue nor calcofluor white staining detected any alteration in infected PB1 sheaths at 1 dpi. Interestingly mode of *R. solani* pathogenesis was found to be quite similar in other rice cultivars (TP309 and TETEP), although the size of disease lesion and extent of cellular disintegration was comparatively less in TETEP (Figs S1, S2). To elucidate the molecular mechanism of susceptible rice *R. solani* interactions, we selected PB1 as a representative rice cultivar and focused on understanding the events associated during transition from 1dpi to 3dpi of pathogenesis.

### Chloroplast localised ROS production in rice during infection

**Figure 2:**
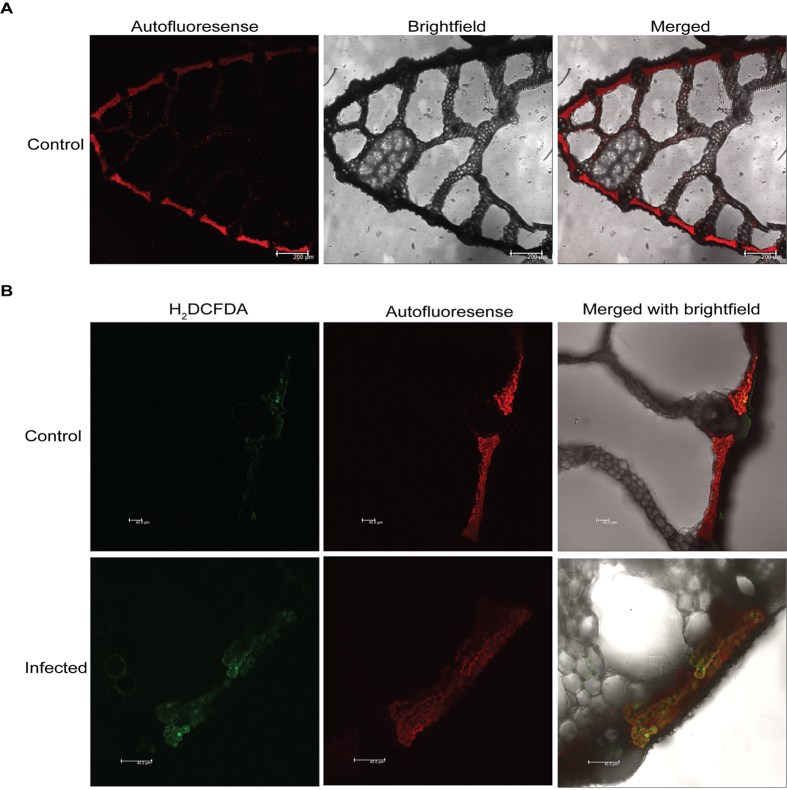
Chloroplast localized ROS in *R. solani* infected PB1 rice. (**A**) T.S. of uninfected rice sheath showing red signal due to chloroplast auto fluorescence. Scale bars = 200 μm. (**B**) Confocal images of T.S of H_2_DCFDA (a green fluorescence dye for ROS) stained 3dpi infected and control rice sheaths. Chloroplast autofluorescence (red) is found to be co-localized with H_2_DCFDA (green) fluorescence in infected sheath while no green fluorescence is visible in control. The data presented in this figure is a representative of at least three independent biological replicates with at least 3 sheaths from different tillers being analyzed at each time points. Scale bars = 40 μm.

We studied the accumulation of reactive oxygen species (ROS) in the infected sheaths. DAB staining reflected ROS to be accumulated in infected lesion adjoining area at 3dpi (Fig. S3A). Cell death was observed at site of lesion as shown in Fig. S3B, infected PB1 rice sheath exhibited trypan blue staining at 3dpi. Dual staining with Evans blue and DAB further suggested overlap between site of ROS production and observed cell death (Fig. S3C). However no visible cell death and ROS accumulation were detected in infected sheaths at 1dpi. To find out if chloroplast is cellular site for ROS production during *R. solani* infection, transverse section of leaf sheaths were stained with H_2_DCFDA and analysed through confocal microscopy. In infected sheaths the green signal due to H_2_DCFDA staining of ROS was found to be co-localized with red autofluorescence signal of chloroplast (Fig. 2).

**Figure 3:**
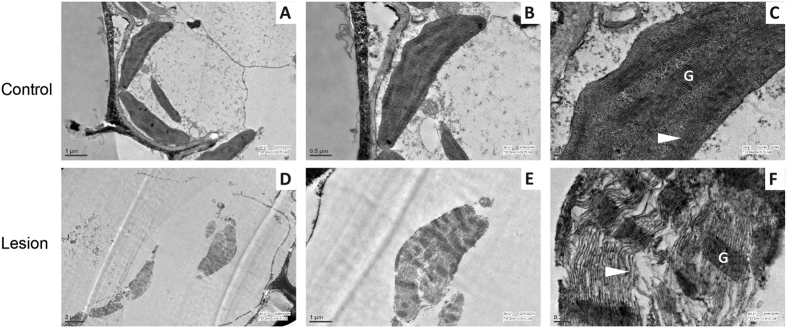
The effect of *R. solani* infection on chloroplast ultrastructure. Chloroplast structure and thylakoid organization in control and infected rice sheath (PB1) were analysed by transmission electron microscopy (TEM). (**A** and **D**) depict cellular view while (**B** and **E**) and (**C** and **F**) represent organellar and ultrastructure view of chloroplast, respectively. Chloroplasts from control sheath do not show alteration (**A**,**B** and **C**) while structural disintegration of chloroplast grana along with de-stacking of thalakoid and stromal lamellae were observed in infected sheath at 3dpi (**D**,**E** and **F**). Arrowhead depicts stroma thylakoids while granum is depicted as G.

Furthermore, through transmission electron microscopy we analysed the ultrastructure of chloroplast in infected tissues at 3dpi. In control sheaths, the chloroplasts were mostly ellipsoid in shape, the grana and stromal lamellae were well developed while thylakoids were arranged densely along the long axis of the chloroplasts (Fig. 3A–C). However in infected sheaths, structural distortion of chloroplast along with de-stacking of thalakoid and stromal lamellae were quite apparent (Fig. 3D–F).

### *R. solani* infection dampens photosynthesis in rice

**Figure 4:**
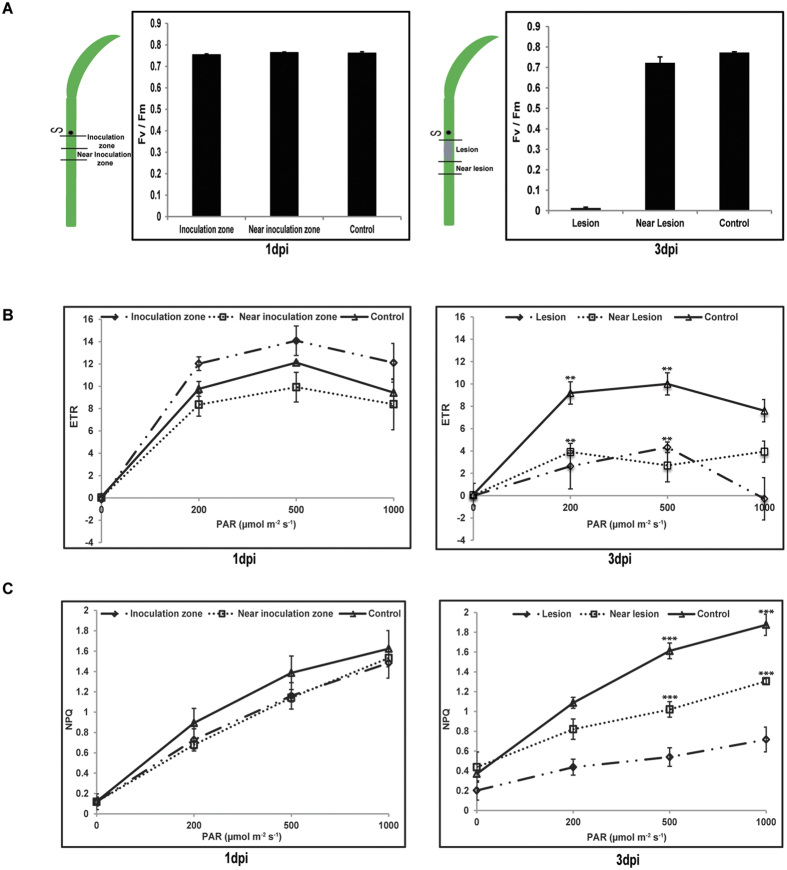
The effect of *R. solani* infection on photosynthetic efficiency of PB1 rice sheath. (**A**) The chlorophyll fluorescence imaging of rice sheath at 1dpi and 3dpi. Schematic diagram of the infected rice sheath depicting zones along with observed maximal photosystem II photochemical efficiency (Fv/Fm) are shown. The electron transport rate, ETR (**B**) and non-photochemical chlorophyll fluorescence quenching, NPQ (**C**) values recorded in different region of rice sheath at 1dpi and 3dpi are depicted. The data presented is representative of at least three independent biological replicates with at least 3 sheaths from different tillers being analysed at each time points. **P < 0.005, ***P < 0.001 represent significant difference estimated by one-way ANOVA.

Photosynthetic performance was measured in different regions of infected rice sheaths at 1dpi and 3dpi. Chlorophyll fluorescence imaging revealed no significant changes in infected and non-infected rice sheaths at 1dpi with respect to photosynthetic parameters like maximum quantum yield of photosystem II (Fv/Fm), electron transport rate (ETR), non-photochemical quenching (NPQ) (Fig. 4A–C). However at 3dpi, when necrotic symptoms become visible, alterations in photochemistry were more pronounced. The Fv/Fm value was almost negligible while the ETR and NPQ values were significantly less in lesion area at 3dpi. Although adjoining lesion area showed no significant difference in Fv/Fm value compared to non-infected control but demonstrated significantly less ETR and NPQ values, particularly at higher irradiance (Fig. 4A–C).

### GC-MS based metabolome analysis revealed major alteration in host machinery

To understand metabolic changes that regulate physiological alterations in rice observed during 1dpi and 3dpi of *R. solani* infection, metabolome analysis was performed using GC-MS (Gas chromatography-mass spectrometry). We selected 50 metabolites that are consistently differentially abundant in rice during pathogenesis of *R. solani*. Out of them 34 were found induced while 16 were decreased in infected sheaths compared to control sheaths during infection transition from 1dpi to 3dpi (Table S1). The differentially regulated metabolites were further classified into different chemical groups and their functions/involvement in different pathways was manually annotated through literature searches. The relative concentration of most of the sugar metabolites (such as sucrose, glucose, fructose, glucosone, galactose, hexopyranose, turanose, maltose and glucopyranose) along with a few fatty acids (fumaric acid and phytol), sugar alcohols (myo-inositol and glycerol 3-phosphate) and phosphoric acid were found decreased at 3dpi compared to 1dpi while majority of organic acids, amino acids, sugar alcohols along with dopamine were found to be abundant at 3dpi (Table 1).

**Figure 5:**
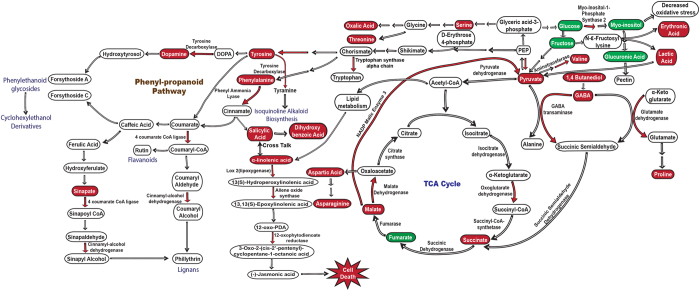
Schematic overview of rice pathways altered during *R. solani* infection. Integrated metabolome and transcriptome analysis reveals major processes that are affected during pathogenesis. The filled boxes represent metabolites identified through GC-MS analysis while the filled arrows indicate differentially regulated genes. The red and green colors represent upregulation and downregulation respectively. The analysis identified increased levels of tyrosine, phenyalanine, dopamine, sinapate metabolites and upregulation of genes such as phenyl ammonia lyase, tyrosine decarboxylase, 4 coumarate CoA ligase and cinnamyl alcohol dehydrogenase associated with phenylpropanoid pathway. Further induction of pyruvate, 1,4 butanediol, γ-aminobutyrate, succinate and malate suggested enhanced respiration as these metabolites are involved in TCA cycle. We also found two phytohormones, salicylate and its sugar conjugate dihydroxybenzoic acid along with jasmonate precursor α-linolenic acid to be induced in the infected sheath. Beside these, some other amino acids such as asparagine, valine, serine and isoleucine were upregulated while threonine and aspartic acid were downregulated. Notably, sugar alcohol myo-inositol and glucuronic acid involved in oxidative stress response and cell wall biosynthesis respectively were found downregulated.

Based upon the abundance of related metabolites it seems that respiratory processes are enhanced in *R. solani* infected rice sheaths at 3dpi (Fig. 5). Several tricarboxylic acid (TCA) cycle intermediates like succinate, pyruvate, aconitate were found induced. Further, we also detected induction of a succinate precursor i.e GABA (γ-amino butyric acid) and a GABA precursor i.e. 1,4-butane diol in infected tissue at 3dpi^20^. Accumulation of aliphatic amino-acids such as proline, valine, isoleucine and threonine were notable. Besides, increased concentration of aromatic amino acids like phenylalanine and tyrosine which potentially serve as precursor molecules for phenylpropanoid pathway suggested host preparedness for biosynthesis of secondary metabolites. Accumulation of phenylpropanoid intermediates such as sinapic acid and dopamine, further confirm the involvement of this pathway during pathogenesis of *R. solani*. α-Linolenic acid, a precursor of JA biosynthesis was highly abundant in infected tissue at 3dpi. In addition, salicylic acid showed increased concentration while a salicylic acid derived sugar conjugate i.e 2, 3-dihydroxybenzoic acid (an inactive form of SA) was found decreased^21^,^22^. Conjointly, organic acids such as oxalic acid (virulence determinant), acetohydroxamic acid (metalloproteinase inhibitor), erythronic acid (acidification of cytoplasm), lactic acid (acidification of cytoplasm), and thiobarbituric acid (peroxidation of lipids) were other notable metabolites which showed accumulation at 3dpi (Table 1).

### The gene expression profiling substantiated the observed metabolomic alterations

To correlate the observed metabolic changes with that of transcriptional alterations, we carried out RNAseq analysis on susceptible rice genotypes (PB1 and TP309) to identify common differentially regulated genes, during transition from 1dpi to 3dpi of *R. solani* pathogenesis. Out of 576 such genes, 550 were up-regulated while 26 were down-regulated (Table S2). These transcripts were mapped onto a virtual rice metabolic pathway using MapMan package to reflect the ongoing metabolic alterations during *R. solani* pathogenesis in rice. The analysis majorly revealed induction of respiration, secondary metabolism, and hormonal alterations (Fig. S4 and Table S3). Respiration seems upregulated as induction of genes involved in glycolysis (phosphoglycerate mutase), fermentation (pyruvate decarboxylase, pyruvate dehydrogenase), TCA cycle (oxoglutarate and malate dehydrogenase, carbonic dehydratase), electron transport chain (NADH dehydrogenase, alternative oxidase) and gluconeogenesis (malate synthase and dehydrogenase, PEP carboxykinase1) were detected. Upregulation of shikimate, non mevalonic acid, terpenoids, phenylpropanoids, phenols, flavonoids and anthocyanin encoding genes advocates for the enhanced secondary metabolism. The upregulation of lipoxygenase (LOX), allene oxidase synthase and 12-Oxophytodienoate reductase (OPR1) highlights the importance of JA signalling during pathogenesis of *R. solani* on rice. Further, *R. solani* infection also resulted in induction of salicylic acid biosynthetic genes (S-adenosylmethionine methyltransferase), ethylene signalling genes (ethylene responsive element binding factor, AP2 domain containing protein, ethylene response factor 1) and auxin related genes (indole-3-acetic acid amido synthase, auxin responsive protein).

Similar to GC-MS analysis, transcripts related to amino acid biosynthesis of tryptophan, tyrosine, proline and cysteine were found to be upregulated during *R. solani* pathogenesis. The pathogenesis also marked an increase in carbohydrate metabolism in the infected tissues (Table S3). In this regard, transcripts encoding invertase (beta-fructofuranosidase; putatively involved in breakdown of sucrose into hexoses) along with biosynthetic genes of sugars like raffinose, trehalose and myo-inositol were upregulated. Furthermore, several genes involved in cell wall biosynthesis (cellulose synthases), modification (expansins) and degradation (pectin esterases) were found to get induced during *R. solani* infection. It is noteworthy that real time PCR analysis also validated the differential expression of few selected genes involved in photosynthesis, secondary metabolism, and hormonal regulation during rice-*R. solani* interactions (Fig. S5).

## Discussion

*R. solani* causes sheath blight disease in rice and is known to significantly damage rice cultivation globally^1^,^23^. Most of the rice varieties are susceptible, however some of them do exhibit moderate to high degree of disease tolerance^19^,^24^. In order to device better strategies to develop durable resistance, it is important to understand the intricacies of sheath blight disease establishment. Using a virulent *R. solani* strain BRS1, we demonstrated that the pathogen is able to follow similar mode of colonization on both susceptible (PB1 and TP309) as well as partially resistant (TETEP) rice cultivars. We observed that initially *R. solani* mycelium grows parallel to veins and upon establishment it forms infection cushion like structures thereby imparting characteristic disease symptoms at 3dpi. Previous studies had also detected formation of infection cushion during pathogenesis of *R. solani*^13^,^25^. Extensive disintegration of cellular anatomy, accumulation of ROS, activation of malformed defense and induction of cell death responses were observed at 3dpi. It seems that *R. solani* might initially avoid causing damage to host tissue, as no alteration was noticed in infected tissues at 1dpi. This might be helpful for the pathogen to escape being recognized by host. However it cannot be ruled out that the delay in visual alteration might be due to the fact that pathogen might need some time to overcome host defense^26^. Upon establishment (3dpi), *R. solani* displayed necrotrophic symptoms, causing extensive cellular damage and death of infected tissues. By adopting integrative transcriptional and metabolomics studies we explored the physiological and cellular changes in rice during infection transition from 1 to 3dpi. Such approach has proven to be helpful in deeper understanding of pathogenicity mechanism of various plant pathogens^27^,^28^,^29^,^30^. Our analysis reflected major metabolic reprogramming including downregulation of photosynthesis, enhanced respiration and accumulation of secondary metabolites in rice during *R. solani* infection.

The necrotic zone of disease lesion not only showed negligible Fv/Fm ratio but also demonstrated low ETR and NPQ values. Inhibition of photosynthesis in both lesions as well as green areas between lesions had been reported previously during necrotrophic phase of beans-*Colletotrichum lindemuthianum* interaction^31^. Although we observed no change in Fv/Fm ratio in area surrounding sheath blight lesion zone in rice, but there was significant decrease in ETR and NPQ value, particularly at higher irradiance. Similar decrease in NPQ value in surrounding lesion had been observed during necrotrophic phases of *Mycosphaerella graminicola* and wheat interactions^32^. It is intriguing why Fv/Fm values (reflecting maximum photochemical efficiency) recorded in near-lesion zone and that of un-infected sheaths are similar at 3dpi while there is decrease in ETR and NPQ value. It is possible that decrease in photosynthetic efficiency during *R. solani* infection may be due to down regulation of photosynthesis associated genes as revealed through real time PCR (Fig. S5). This is in accordance to earlier studies wherein a decrease in photosynthesis, has been reported during both compatible/incompatible interactions with biotrophic as well as necrotrophic pathogens^33^,^34^,^35^,^36^,^37^,^38^,^39^. Downregulation of photosynthesis might lead to changes in carbohydrate metabolism. Both RNAseq and metabolite profiling suggested alterations in level of carbohydrates in *R. solani* infected tissues. Such fluctuations have been reported as plant response against *R. solani* invasion in potato^40^.

Host respiratory machinery including TCA cycle was found upregulated during *R. solani* infections, as apparent in both transcriptome and metabolome data. Plants generally increase respiration to produce energy for manufacturing and mobilizing defence compounds or to meet elevated metabolic demands imposed by pathogen attack^34^,^41^. Such enhanced respiration for prolonged duration might result in generation of ROS^42^. Indeed we observed enhanced ROS accumulation in infected rice sheaths. Also the role of ROS during *R. solani* and wheat interaction has been demonstrated in a recent report^43^. Chloroplast is known to be associated with ROS production during stress^44^,^45^,^46^. Our data suggests that chloroplast is the site of ROS production in *R. solani* infected sheaths, as the ROS staining and chloroplast auto-fluorescence coincided. Enhanced ROS accumulation in chloroplast might be linked with alteration in ultrastructure of grana and thylakoids in the infected rice tissues as observed in this study. Furthermore, it seems that the accumulation of ROS may be a pre-requisite for necrotrophic cell death in the infected tissues (Fig. S3).

Hormonal signalling seems to be altered during susceptible rice-*R. solani* interactions. The upregulation of JA biosynthesis genes and precursor metabolite (α-linolenic acid) suggest the induction of JA signalling^47^. Similar to our data, a previous study had also reported upregulation of LOX gene (involved in JA biosynthesis) during pathogenesis of *R. solani* in rice^48^. Beside JA, enhanced accumulation of SA was detected in infected tissues. This shows that both JA and SA signalling might be altered during pathogenesis of *R. solani* and a cross-talk between them might be functional^49^,^50^. However further research is needed to explore the importance of JA-SA signalling and their potential cross-talks during establishment of rice sheath blight disease. Moreover, our study suggests activation of secondary metabolic pathways in rice sheaths, as reflected by both GC-MS and gene expression analysis. The present study, by integration of transcriptomic and metabolomic data highlights the induction of phenylpropanoid pathway during *R. solani* infections (Fig. 5). The increased biosynthesis of aromatic amino acids like Phe, Tyr and Trp observed during *R. solani* infections might be helpful in this regard. Alteration in secondary metobolism had been also reported previously during *R. solani* infection^51^,^52^,^53^,^54^. GC-MS study revealed several other metobolites to be alterated in rice during *R. solani* infection (Fig. 5). The increased erythonic acid content of infected tissue can be associated with decrease in photosynthetic efficiency^55^,^56^. The downregulation of myo-inositol might promote enhanced ROS accumulation in infected tissues, as it acts as antioxidant to reduce oxidative stress^57^. Another metabolite glucuronic acid, involved in myo-inositol oxygenation pathway was found to be downregulated. Glucuronic acid is known to be involved in biosynthesis of hemicellulose and pectin polymers^58^. This along with transcriptome data suggested that cell wall biosynthesis machinery of the host might get curtailed during *R. solani* pathogenesis and this in turn might facilitate host penetration by pathogen.

**Figure 6:**
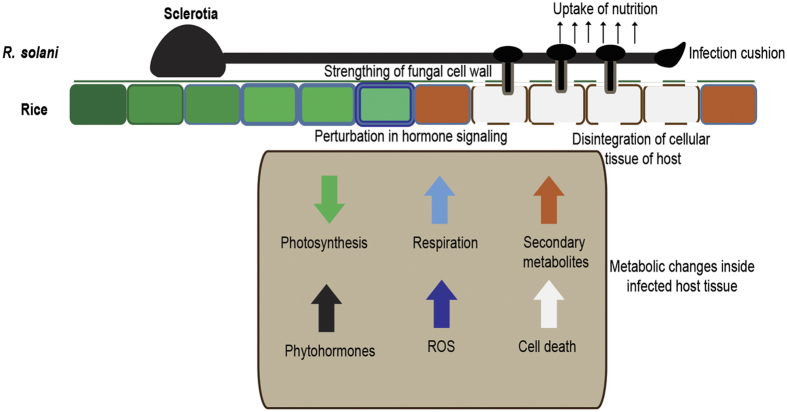
Model of molecular processes underlying rice-*R. solani* interaction. Upon inoculation *R. solani* sclerotia germinate along rice veins and form infection cushion during disease establishment. Downregulation of photosynthesis and increased respiration, secondary metabolism, phytohormones, ROS accumulation and cellular disintegration leading to host cell death were noteworthy changes during pathogenesis. Upward and downward arrow represents upregulation and downregulation of genes/processes, respectively.

The present study describes the pathological, physiological, and integrated metabolome-transcriptome analysis to understand the intricacies of susceptible rice-*R. solani* interactions. Novel insights about metabolic perturbation has been unfolded and a model summarising the interactions is being proposed (Fig. 6). *R. solani* pathogenesis leads to downregulation of photosynthesis, increased respiration and secondary metabolism, ROS accumulation and cell death in rice. It would be interesting to explore how such perturbations promote disease susceptibility against this necrotrophic pathogen and exploit them in developing disease resistance.

## Experimental Procedures

### Biological materials and pathological assays

*Oryza sativa* ssp. *indica* (cv. PB1 and Tetep), *O. sativa* ssp. *japonica* (cv. TP309) and *R. solani* AG1-IA isolate BRS1 were used in this study. BRS1 sclerotia were cultured on PDA (39 g/L; Potato Dextrose Agar; Himedia, Mumbai, India) plates at 28 °C. The freshly prepared equal sized sclerotia were used for inoculation. Rice was grown in a PGV36 conviron plant growth chamber at 28 °C temperature, 80% relative humidity and 16/8 hr of day/night. 60 days old rice sheaths were infected with *R. solani* sclerotia, following previously described procedure^19^. After 1 , 2 and 3days post inoculation (dpi), the infected tissues (including 1 cm up and down from the site of infection) were harvested and assessed for microscopic, metabolomics and expression analysis.

### Staining and microscopic analysis

To study pathological development of *R. solani*, rice leaves were stained with 0.1% calcofluor white M2R (Sigma Aldrich) in 0.05 M PBS (pH 8.5) for 30 minutes and washed twice with MQ water. Samples were observed under DAPI filter in Laser Scanning Microscope (AOBS TCS-SP2, Leica, Germany) using 20X objective. The depth of optical sections ranged from 50–70 μM and images were analysed using LAS AF Version: 2.6.0 build 7266 software.

For microtome sectioning, the infected and control rice sheaths were immersed in FAA fixative (3.7% v/v formaldehyde (Fisher Scientific Chemicals), 50% ethanol (Fisher Scientific Chemicals), 5% acetic acid (Fisher Scientific Chemicals) and subjected to vacuum until the tissue pieces sank. After overnight incubation at 4 °C, the tissues were passed through different concentration (30%, 50%, 70%, 95% and 100%; 2 hrs each step) of tert-butanol (Fisher Scientific Chemicals) for dehydration. Following this, samples were embedded in Paraplast Plus (Sigma Aldrich Chemicals) and sectioned (15 μm) on a rotary microtome (Leica RM2265). Sections were fixed onto polylysine (Sigma Aldrich Chemicals) coated glass slides (GEM Slides) at 40 °C water bath. Tissue sections were de-waxed in xylene (Fisher Scientific Chemicals) and were stained in either toluidine blue (Himedia) solution (0.01%, dissolved in MQ water) for 10 minutes or 0.1% calcofluor white M2R (Sigma Aldrich) solution (in 0.05 M PBS; pH 8.5) for 30 minutes. Toluidine blue stained sections were observed under white light while calcofluor white stained sections were observed using a stereo zoom microscope (Nikon AZ100) coupled with Nikon camera (Nikon Digital Sight DS-Ri1). All microscopic analyses were performed with at least three biological replicates and consistent results were obtained.

### ROS production and cell death assays

To visualize ROS accumulation DAB (3,3′-Diaminobenzidine) staining were performed. The infected sheaths were excised, stained with 1 mg/mL DAB (prepared in 50 mM Tris acetate buffer, pH 5.0), and incubated at 25 °C for 24 h in dark. Following staining, sheaths were boiled in 95% (v/v) ethanol for 20 min to remove chlorophyll and rehydrated in 40% (v/v) glycerol. H_2_O_2_ was visualized as a brown colour due to DAB polymerization under Nikon stereo-zoom microscope with optical filters at 488 nm excitation and 505 nm emissions.

Trypan blue staining was used for cell death assay. Rice leaf sheaths were stained in 0.01% solution of trypan blue (Himedia) and incubated overnight with shaking at low speed. Following washing, the sheaths were destained in choral hydrate solution for 24 hrs with slow shaking and observed in bright field under Nikon stereo zoom microscope (Nikon AZ100) coupled with Nikon camera (Nikon Digital Sight DS-Ri1). Simultaneous detection of H_2_O_2_ accumulation and cell death was performed by incubation of DAB-infiltrated sheath in dark for 24 hours followed by 2 hrs of light incubation. Upon this, the sheaths were vacuum infiltrated with 1.3 mM Evans blue (Himedia) solution (0.125 g dissolved in 100 mL of distilled water). After 15 min of incubation in dark, leaves were boiled in a clearing solution (3:1, ethanol:glacial acetic acid) for 15 min. The samples were mounted with 60% glycerol (Fisher Scientific Chemicals), and observed in bright field under Nikon stereo zoom microscope (Nikon AZ100) coupled with Nikon camera (Nikon Digital Sight DS-Ri1). Each experiment was repeated three times, with atleast 5 infected sheaths being analysed each time.

### Localization of ROS

To study the localization of ROS in the infected samples, T.S. of the rice sheaths were cut manually and stained with fluorescent stain H_2_DCFDA (2′,7′-dichlorodihydrofluorescein diacetate). 100 mM stock solution of H_2_DCFDA (Molecular Probes, Invitrogen) was prepared by dissolving H_2_DCFDA in dimethyl sulfoxide. The sections were incubated in 5 *μ*M working concentration of H_2_DCFDA for 2 min and observed under GFP filter of a laser scanning microscope (AOBS TCS-SP2, Leica, Germany) using 20X objective. The images were analysed using LAS AF Version: 2.6.0 build 7266 software.

### Transmission electron microscopy

Control and infected leaf sheath tissue were cut approximately into 5 × 2 mm, and were immediately fixed in 2.5% glutaraldehyde in 0.1 M phosphate buffer (pH 7.2) for 12 h (6 °C). After rinsing with phosphate buffer, tissues were fixed with 2% osmium tetraoxide in sodium phosphate buffer. Dehydration was accomplished by gradual ethanol series and tissues were embedded in epoxy resin. Ultrathin sections (800 nm) were stained with uranyl acetate and lead citrate. The sections were viewed and photographed with TECNAI 200 Kv TEM (Fei, Electron Optics) at SAIF -EM facility unit at All India Institute of Medical Sciences (AIIMS), New Delhi, India.

### Chlorophyll fluorescence imaging

Chlorophyll fluorescence readings were taken using portable photosynthesis system LI-6400 XT (LI-COR Biosciences, Inc.) in healthy and infected sheaths at 1dpi and 3dpi. A measuring area of 16mm × 5mm was taken for considering maximum spatial resolution in different zones of infected and healthy sheaths. Under saturating pulse, maximum quantum yield (Fv/Fm ratio) was measured from dark adapted leaves after 12 hours of night cycle. Under actinic light, the relative rate of electron transport rate was calculated as ETR = 0.5 × leaf absorptance × *ϕ*_PSII_PAR. Non-photochemical quenching (NPQ) was calculated by the formula: NPQ = (F_m_ − F_m_´)/F_m_´. The fluorescence parameters measured were at different PAR absorptivity, i.e. 0, 200, 500, 1000 μmol (photons) m^−2^ s^−1^.

### GC-MS analysis

The GC-MS metabolite profile was obtained from 3 biological replicates of *R. solani* infected and non-infected samples, at 1dpi and 3dpi. 300 mg of tissues from each sample (pooled from 3 different sheaths) was crushed in liquid N_2_ and homogenized in 1.5 ml of methanol (HPLC grade) with 60 μl of 0.2 mg/ml adonitol (Sigma Aldrich). The samples were incubated for 15 mins at 70 °C and thereafter 1.4 ml of water along with 750 μl of chloroform were added. Upon vortexing and centrifugation at 2200 g for 5 mins, supernatant was isolated and vacuum dried for 8–12 hrs. The dried residue was derivatized in 40 μl of methoxyamine hydrochloride (20 mg/ml in pyridine) for 90 mins in a water bath at 37 °C. Trimethylsilylation was performed by addition of 60 μl of MSTFA (Sigma Aldrich) and incubation at 37 °C for 30 mins. The GC-MS instrument consisted of an autosampler-autoinjector (AOC-20si), gas chromatograph-mass spectrometer (Shimadzu QP2010 Ultra). The analysis was conducted in a splitless mode using 2 μl as injection volume. Gas chromatography was performed using Rtx-5MS column (Restek Corporation, US) and helium as carrier gas. The temperature program comprised of 80 °C isothermal heating for 2 mins, followed by ramp rate of 5 °C/min to 250 °C withhold time 2 mins and a final ramp rate of 10 °C/min to 280 °C withhold time 14 mins. The chromatograms and mass spectra were analysed using GCMSsolution software (Shimadzu). The spectral libraries used for peak identification were NIST8 and WILEY8. Peak normalization and statistical analysis was performed in Excel macro tool (Tool for statistical analysis on Microsoft Excel, Platform for RIKEN Metabolomics)^59^. The peak area obtained for each of the metabolite was normalized with internal control (i.e. Adonitol). Considering that the missing value of a metabolite in a particular sample might be due to its level below detection limit of the instrument, we replaced it by a minimal value (0.000001). As described in recent protocols, the reproducibly detected metabolites in each of the three replicate were selected and the median value were used for estimation of their differential accumulation in infected and control samples^60^,^61^. The mean value of metabolites were calculated by comparison between 3dpi and 1dpi, for both infected and control samples. The log fold change between infected and control samples is reflected in Table S1.

### Transcriptome sequencing and differential expression analysis

RNAs were isolated from *R. solani* infected sheaths of susceptible PB1 and TP309 rice genotypes (5 sheath samples pooled up) at 1dpi and 3dpi using RNeasy Plant Mini Kit (Qiagen). Transcriptome sequencing was performed using Paired end (PE) 2 × 100 bp library on Illumina HiSeq 2000. The library was prepared using TrueSeq stranded mRNA HT sample preparation kit. TrueSeq PE cluster kit v3-cBot-HS was used for cluster generation, while the TrueSeq SBS kit v3-HS (200 cycles) was used for RNA sequencing. All these protocols were performed using standard protocols recommended by the manufacturer (Illumina Inc.). The average quality score of sequence reads obtained through RNAseq analysis in different samples are summarized in Fig. S6. Trimmomatic version-0.32 was used for pre-processing of reads to remove TruSeq3 pair-end adapter sequences^62^. Using Bowtie2 version 2.0.6 alignment tool^63^, clean reads were mapped onto the available indica and japonica rice genomes, independently for all samples. The cleaned rice reads were used for sequence assembling using Trinity version r20140717^64^. Transdecoder version r20140704^64^ was used to detect coding regions. Annotation was performed by using blastx, blastp, hmmscan, and rnammer tools. An e-value cut-off of 1e^-6^ was applied for all blast searches. RSEM v1.2.19 was used for differential gene expression analysis^65^. This program aligns input reads against a reference transcriptome with Bowtie2 and calculates expression values using alignments. EBSeq program was used to perform differential expression analysis. The common amongst differentially regulated transcripts with FDR less than 0.05 in both the susceptible rice genotypes were selected for further analysis. MapMan package^66^ was used to visualize alteration in metabolic pathway.

### Real time qPCR analysis

The expression dynamics of selected rice genes (Table S4) were analysed during 1dpi and 3dpi of *R. solani* infection on susceptible cultivar PB1. PCR primers were designed for each of these genes in such a manner that they do not amplify any fungal sequences. cDNA synthesis was done using Verso cDNA synthesis kit (Thermo Fisher Scientific Inc.) as per specified protocol. 2 μg of RNA were used cDNA synthesis and was diluted 5 times for real time PCR. The Ct values of the genes were normalized from the reference gene (i.e. 18 s rDNA) following which the 1dpi and 3dpi infected samples were adjusted from control samples. The difference in the Ct values of 3 versus 1dpi was used to estimate the log fold change. All experiments were performed with at least three biological and technical replicates.

## Additional Information

**Accession codes**: The Illumina sequence data from this study have been submitted as BioProject ID: PRJNA298635 to the NCBI Sequence read archive.

**How to cite this article**: Ghosh, S. *et al*. Alterations in rice chloroplast integrity, photosynthesis and metabolome associated with pathogenesis of *Rhizoctonia solani. Sci. Rep.* **7**, 41610; doi: 10.1038/srep41610 (2017).

**Publisher's note:** Springer Nature remains neutral with regard to jurisdictional claims in published maps and institutional affiliations.

## Supplementary Material

Supplementary Data

Supplementary Dataset 1

Supplementary Dataset 2

Supplementary Dataset 3

## Figures and Tables

**Table 1:** Metabolites altered during pathogenesis of *R. solani* in PB1 rice.

Metabolite	Function/Pathway	Log fold change (Inf. vs Cont.)
**Sugar**		
Glucopyranose	Isomer of glucose	0.575
Hexopyranose	Isomer of glucose	−0.009
Galactose	Storage sugar	−0.037
Maltose	Glycolysis	−0.149
Sucrose	Sugar transportation	−0.187
D-Fructose	Glycolysis	−0.556
D-Turanose	Isomer of sucrose	−0.793
D-Glucose	Glycolysis	−5.920
4-Ketoglucose/D-Glucosone	Storage sugar	−10.392
**Organic acids**		
Pyruvic Acid	Glycolysis/TCA cycle	4.912
cis-Aconitate	TCA cycle	4.536
Cyanuric acid	Unknown	4.414
Isobutyric acid	Catabolism of valine	4.066
Acetohydroximic acid	Inhibitor metalloproteinase	3.675
Oxalic Acid	Virulence determinant	0.741
Succinic acid	TCA cycle	0.657
Erythronic acid	Cytoplasm acidification	0.524
Lactic acid	Cytoplasm acidification	0.301
Thiobarbituric Acid	Substrate for lipid peroxidation	0.281
4-Biphenylcarboxylic acid	Component of jarin, JA inhibitor	0.232
Salicylic acid	Defense hormone	0.046
Sinapic Acid	Phenylpropanoid pathway	0.012
Malate	TCA cycle	0.011
Dihydroxybenzoic acid	Degradation product of salicylic acid	−0.042
D-Glucuronic acid	Synthesis of hemicellulose, pectin polymer	−0.072
Talonic Acid	Oxidised monosaccharides	−9.471
**Fatty acids**		
α-Linolenic acid	Defense hormone JA biosynthesis	5.258
Palmitic acid	Cell membrane component	0.792
Fumaric acid	TCA cycle	−3.886
Phytol	Chlorophyll breakdown product	−4.703
**Amino acids**		
Tyrosine	Phenylpropanoid pathway	5.098
L-Asparagine	Nitrogen storage and transport	1.361
L-Proline	Endurance during stress	1.146
Phenylalanine	Phenylpropanoid pathway	0.776
I-Aspartic Acid	Pyrimidine metabolism	0.393
I-Valine	Component of elicitins	0.358
γ-Aminobutyric acid	Precursor of GABA	0.292
Serine	Biosynthesis of phospholipids & sphingolipids	0.178
L-Valine	Component of elicitins	0.145
L-Isoleucine	Accumulates during resistant interaction	0.144
L-Threonine	Confers resistance against pathogen	0.133
L-Aspartic Acid	Pyrimidine metabolism	0.109
**Sugar alcohols**		
D-Myo-Inisitol	Unknown	4.659
D-Glucitol	Storage sugar	0.718
1,4-Butanediol	Precursor of succinate	0.688
Ethanolamine	Cell membrane component	0.041
Myo-Inositol	Decrease oxidative stress	−0.619
Glycerol 3-phosphate	Amino acid biosynthesis	−4.369
**Others**		
Dopamine	Phenylpropanoid pathway	5.470
Phosphoric acid	Source of phosphate	−0.335
