# Supplementary material for: Alterations in rice chloroplast integrity, photosynthesis and metabolome associated with pathogenesis of Rhizoctonia solani
Source: Sci Rep. 2017 Feb 6;7:41610. doi: 10.1038/srep41610 (PMC5292701; doi:10.1038/srep41610)
Supplement: Supplementary Data [file srep41610-s1.pdf]

Alterations in rice chloroplast integrity, photosynthesis and metabolome associated with pathogenesis of *Rhizoctonia solani*

Srayan Ghosh[1#], Poonam Kanwar[1#], Gopaljee Jha[1]*

[1:] Plant microbe interactions laboratory,

National Institute of Plant Genome Research,

Aruna Asaf Ali Marg, New Delhi-110067, India

[#]: equal contribution

[*]: to whom correspondence should be addressed

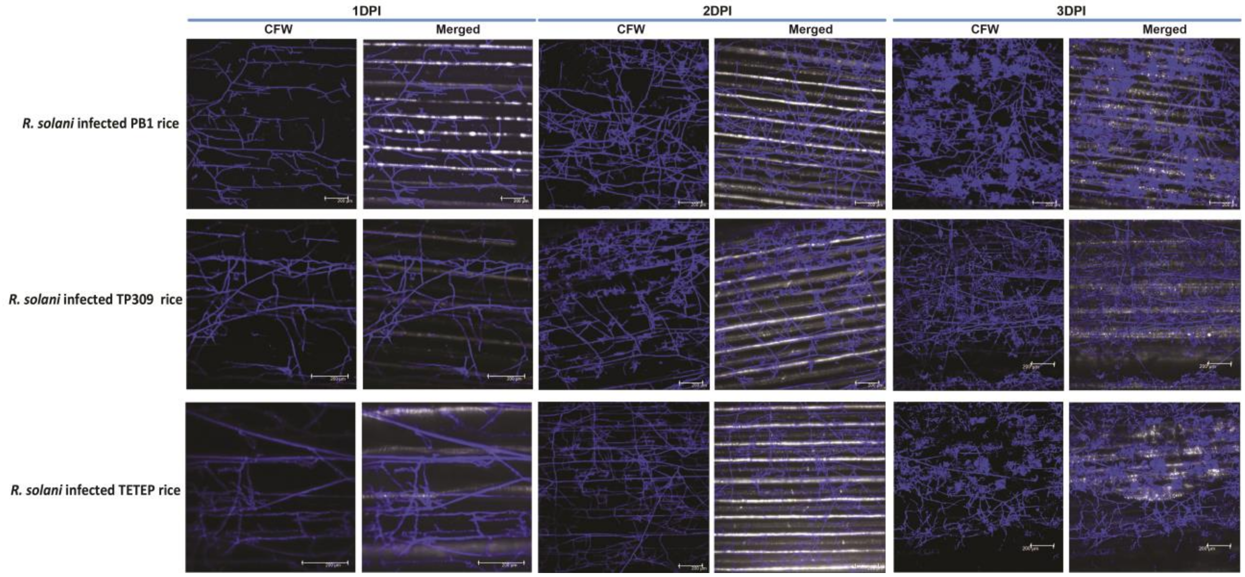

Fig. S1. Pathogenic development of *R. solani* on different rice cultivars. Confocal images of calcofluor white (CFW) stained infected PB1, TP309 and TETEP rice sheaths at different time points. At 1dpi, germinating fungal hyphae were growing parallel to rice veins. By 2dpi increased hyphal branching and mycelial mass were observed. At 3dpi formation of infection cushions coinciding with necrotic disease lesions were observed. At each time point, the first panel reflects images obtained in DAPI filter while the second panel reflects merged bright field images. Scale bar = 40 µm.

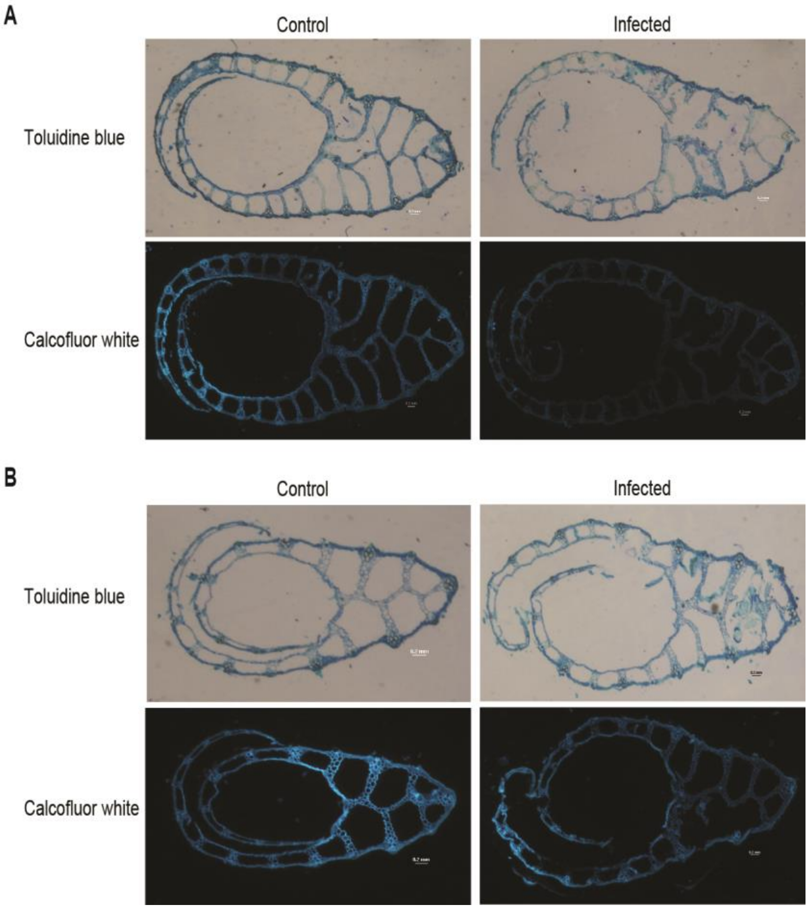

Fig. S2. T.S of toluidine blue and calcofluor white stained *R. solani* infected TP309 (A) and TETEP (B) rice sheaths. At 3dpi, the infected sheaths demonstrated distorted cellular anatomy and staining pattern were faint. However, proper staining of infected sheaths without any anatomical alteration was observed at 1 dpi. Scale bar = 0.2mm.

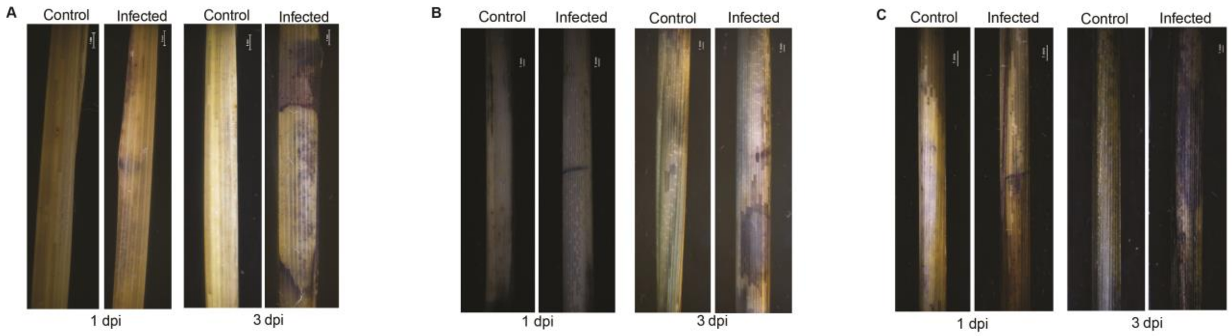

Fig. S3. ROS accumulation and induction of cell death responses in PB1 rice upon *R. solani* infection. (A) Microscopic images of DAB stained rice sheath, brown coloration signifies ROS accumulation in infected tissues. (B) Microscopic images of trypan blue stained PB1 rice sheath, blue coloration signifies cell death in infected sheaths. (C) Dual staining of infected PB1 sheaths with DAB and Evans blue reflecting ROS accumulation (brown) overlapping with observed host cell death (blue) in lesion area. Scale bar = 1mm.

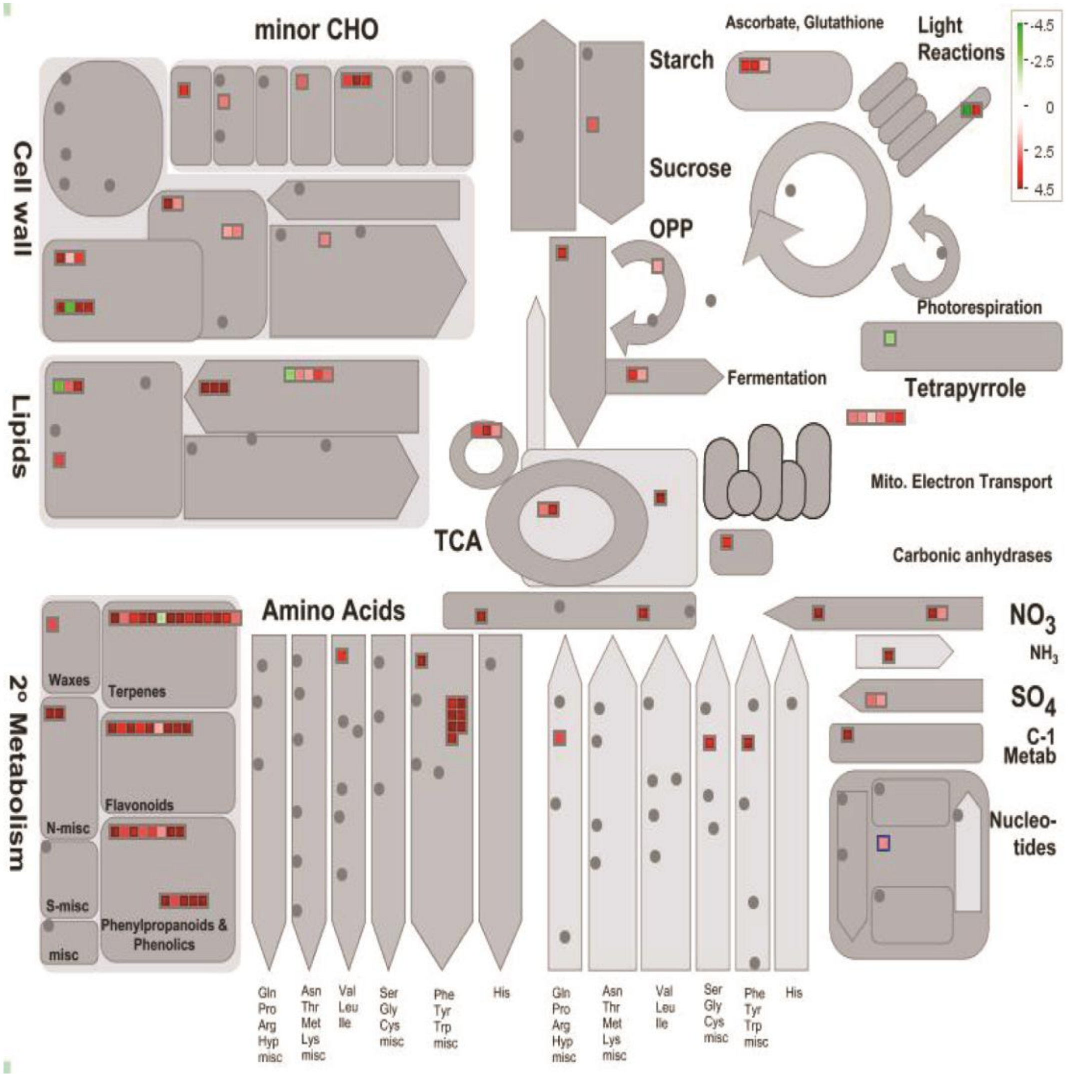

Fig. S4. MapMan based metabolic overview of differentially regulated rice genes during susceptible interactions.

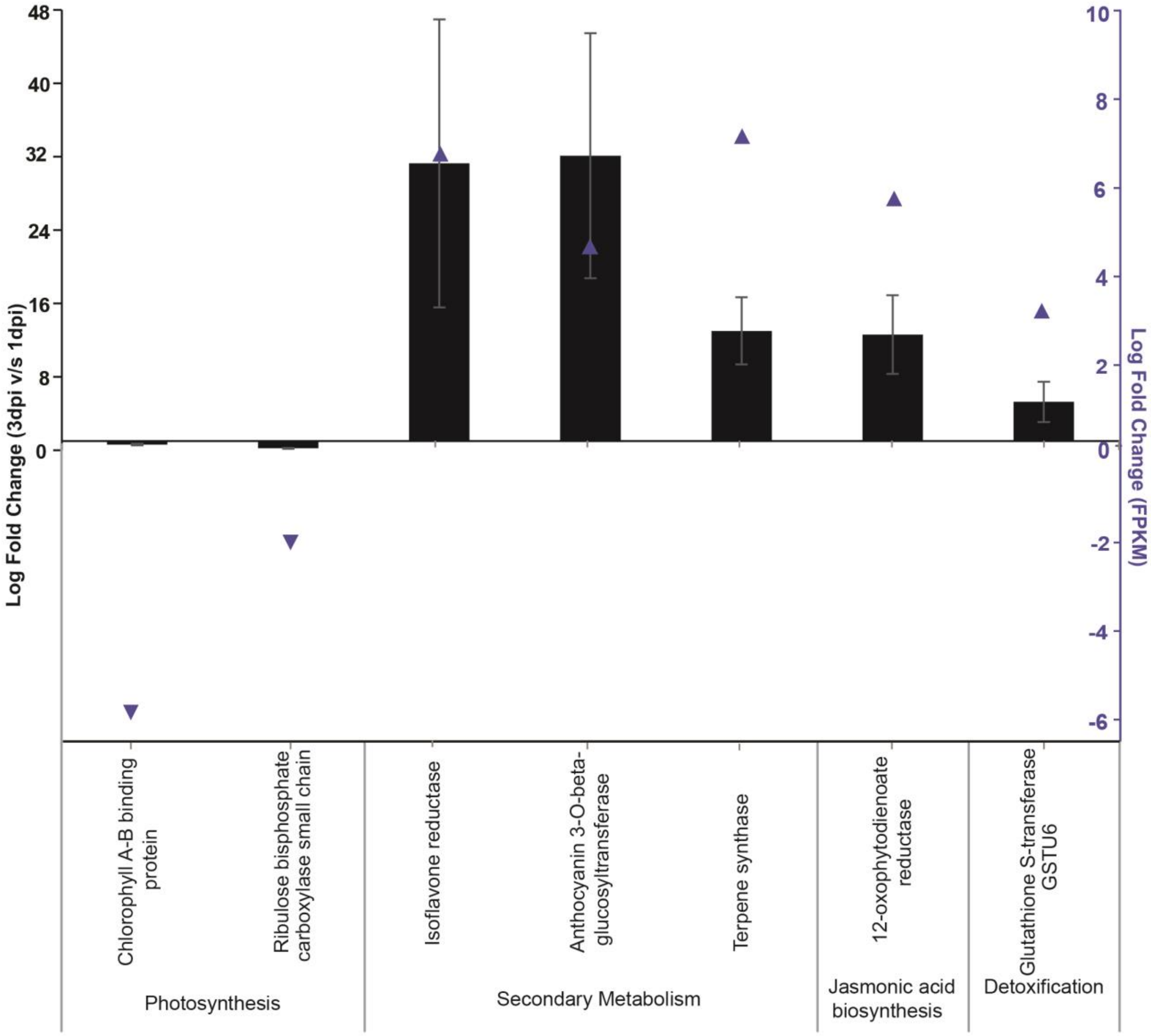

Fig. S5. qRT-PCR based relative expression quantification of selected rice genes during pathogenesis of *R. solani* on PB1 rice. Primary Y axis (black) shows log fold change calculated from real time data, whereas secondary Y axis (blue) indicates log fold change calculated from FPKM values from RNAseq data.

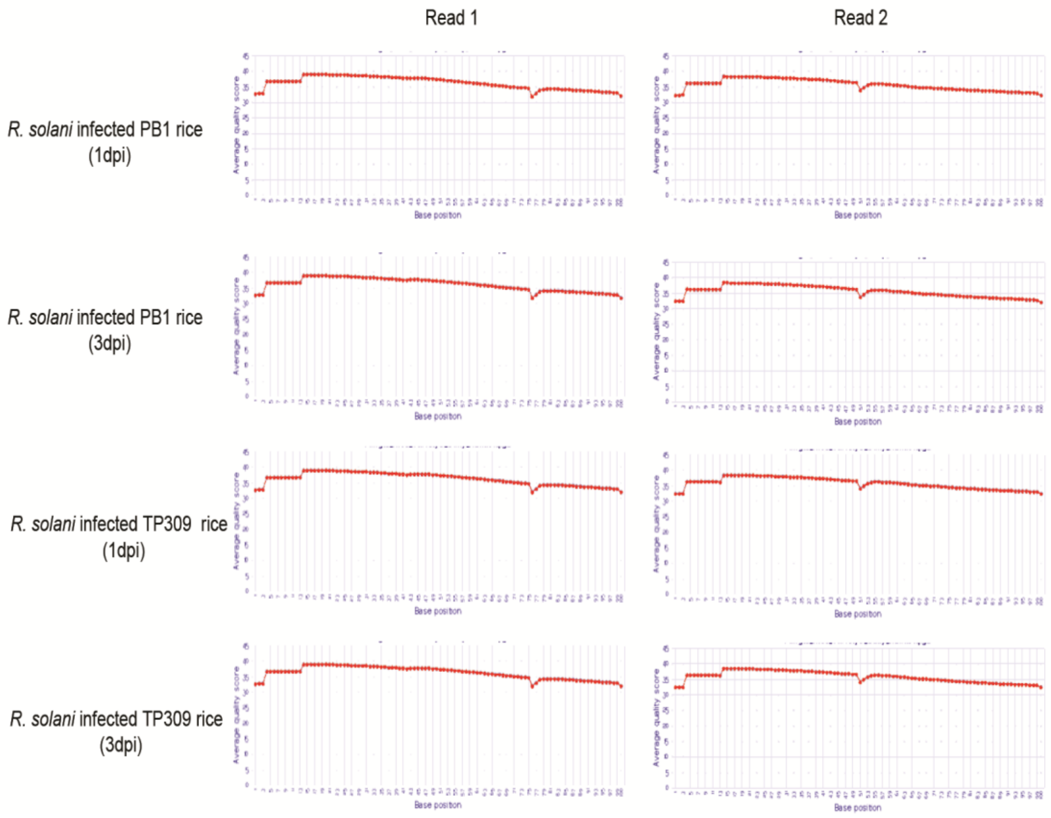

Fig. S6. Graph showing average quality score of RNAseq reads.

Table S4. Primer sequences used for qPCR based expression analysis.

**Primers**	**Locus Id**	**Gene Name**	**Nucleotide sequence 5'- 3'**
OsIFR_RTF	LOC_Os01g13610.1	Isoflavone reductase	TCGTGGACGAGAAGGACATG
OsIFR_RTR			CCGCACGTACAGGATCTTGTT
OsOPR_RTF	LOC_Os01g62480.1	12-oxophytodienoate reductase	AGGGTACGACCGGGAAGAAG
OsOPR_RTR			CCAAGAAGAGCCTCCCATAGG
OsAGT_RTF	LOC_Os03g24690.1	Anthocyanin 3-O-beta-glucosyltransferase	TGGGAGAAGTTCCAGAGCTTAGA
OsAGT_RTR			GCGGTAGTGCTCGACGTAGTC
OsTPS_RTF	LOC_Os04g47720.1	Terpene synthase	TACTCTCGTGCGCGAATCAT
OsTPS_RTR			GTGGCGTGGATATCATAGATGTCA
OsLHC_RTF	LOC_Os05g10650.1	Chlorophyll A-B binding protein	CGCGGAGCTCAAGGTGAA
OsLHC_RTR			CGATGGCCTGGACGAAGA
OsGSTU6_RTF	LOC_Os07g24190.1	Glutathione S-transferase GSTU6	AGCGCCTACGTCACCAGAGT
OsGSTU6_RTR			GTCGCTCTTGTTGCGGAGAT
OsRubisc_RTF	LOC_Os07g26540.1	Ribulose bisphosphate carboxylase small chain	TTCAGCAAGGTCGGATTCGT
OsRubisc_RTR			CATGGGCAGCTTCCACATG
Os_18sF	18s rRNA		CTACGTCCCTGCCCTTTGTACA
Os_18sR			ACACTTCACCGGACCATTCAA
